# Die MIRAGE-Studie (MR-Linac-basierte Körperstereotaxie bei Prostatakarzinomen) – Precision at its best?

**DOI:** 10.1007/s00066-023-02194-3

**Published:** 2024-01-05

**Authors:** Felix Ehret

**Affiliations:** 1 Department of Radiation Oncology, Charité – Universitätsmedizin Berlin, Corporate Member of Freie Universität Berlin and Humboldt-Universität zu Berlin, Berlin, Germany; 2 Charité – Universitätsmedizin Berlin, Berlin, Germany; German Cancer Consortium (DKTK), partner site Berlin, and German Cancer Research Center (DKFZ), Heidelberg, Germany

## Hintergrund

Die Körperstereotaxie („stereotactic body radiotherapy“ [SBRT]) findet vermehrt Einzug in die Behandlung des Prostatakarzinoms (PCa) [1]. Durch den Einsatz von Magnetresonanztomographie(MR)-gestützten Linearbeschleunigern (MR-Linac) und deren potenzielle Vorteile unter anderem hinsichtlich der intra- und interfraktionellen Dosisapplikation erhofft man sich eine Zunahme der Konformität und damit Reduktion an Nebenwirkungen durch die Möglichkeit der Herabsetzung von Sicherheitssäumen [2]. Die MIRAGE-Studie prüft daher die Hypothese, ob eine Reduktion des Sicherheitssaums bei MR-Linac-gestützter SBRT des PCa zu einer verminderten urogenitalen Akuttoxizität führt [3].

## Patienten und Methodik

Die MIRAGE-Studie ist eine prospektive, randomisierte, zweiarmige Phase-III-Interventionsstudie, welche monozentrisch an der University of California, Los Angeles, USA, durchgeführt wurde. Patienten im Kontrollarm (1:1-Randomisierung) erhielten eine SBRT mit Planung mittels klassischer Computertomographie (CT) und 4 mm Sicherheitssaum für das Planungszielvolumen (PTV). Im experimentellen Arm erfolgte eine SBRT mittels MR-Linac und die Sicherheitssäume beim PTV wurden auf 2 mm reduziert. Alle Patienten (in beiden Armen) erhielten eine SBRT mit 5 × 8 Gy. Der primäre Endpunkt der Studie war die Inzidenz an akuten urogenitalen Grad-≥ 2-Nebenwirkungen innerhalb von 90 Tagen nach Durchführung der SBRT gemäß den Common Terminology Criteria for Adverse Events (CTCAE) Version 4.03. Gemäß Studienprotokoll wurde eine Reduktion der Toxizität von 29 % auf 15 % zum Vorteil des experimentellen Studienarms angenommen. Hierfür benötigt es pro Studienarm 150 Patienten, um eine statistische Power von 83,7 % zu erreichen. Es erfolgte eine geplante Interimsanalyse zur Nichtwirksamkeit der Intervention nach 100 eingeschlossenen Patienten, die eine Nachbeobachtungsdauer von mindestens 90 Tagen hatten. Sekundäre Endpunkte der Studie umfassen „patient-reported outcomes“ (PRO) mittels International Prostate Symptom Score (IPSS) und Expanded Prostate Cancer Index Composite-26 (EPIC-26).

## Ergebnisse

Im Rahmen der Analyse für den primären Endpunkt wurden insgesamt 154 Patienten eingeschlossen, davon 78 Patienten im experimentellen Arm. Die Inzidenz akuter urogenitaler Nebenwirkungen Grad ≥ 2 in der MR-Linac-Gruppe war signifikant niedriger als in der Kontrollgruppe (24,4 % vs. 43,4 %, *p* = 0,01). Dieser Vorteil zeigte sich auch bei der Inzidenz der akuten gastrointestinalen Grad-≥ 2-Nebenwirkungen (0,0 % vs. 10,5 %, *p* < 0,01). Diese Reduktion an Nebenwirkungen spiegelte sich auch positiv in den PRO wider. So war der Anteil an Patienten im experimentellen Arm, die einen Anstieg um mindestens 15 Punkte im IPSS einen Monat nach SBRT vorwiesen, im Vergleich zur Kontrollgruppe geringer (6,8 % vs. 19,4 %, *p* = 0,01). Zusätzlich gab es in der MR-Linac-Gruppe weniger Patienten, die einen als klinisch signifikant definierten Punktzahlabfall in der gastrointestinalen EPIC-26-Domäne hatten (25,0 % vs. 50,0 %, *p* < 0,01). Die Grenze für diesen relevanten Abfall wurde beim EPIC-26 bei 12 Punkten festgesetzt.

## Schlussfolgerungen der Autoren

Die Autoren folgern, dass eine Reduktion von Sicherheitssäumen mittels MR-Linac zu einer klinisch messbaren und relevanten Verringerung akuter Nebenwirkungen sowohl urogenitaler als auch gastrointestinaler Natur führt. Zusätzlich spiegeln sich diese Vorteile auch in den PRO wider. Abschließend weisen die Autoren darauf hin, dass es noch einer längeren Nachbeobachtung bedarf, um die langfristigen Vorteile der Behandlungstechnik abschließend evaluieren und bewerten zu können.

## Kommentar

Technische Innovationen spielen in der Strahlentherapie und Radioonkologie eine zentrale und wegweisende Rolle. Diesbezüglich ist auch die Entwicklung des MR-Linac zu nennen, der durch verschiedene technische Vorteile gegenüber der klassischen, CT-gestützten Bestrahlung aufwartet [2]. Während die technischen Vorzüge theoretisch auf der Hand liegen, bedarf es jedoch bedeutend mehr, diese im Rahmen randomisierter Studien mit sinnvollen und relevanten Endpunkten in den klinischen Alltag zu überführen. Die MIRAGE-Studie hat sich nun mit dem PCa einer der epidemiologisch und radioonkologisch wichtigsten Tumorentitäten zugewandt, um diesen Beweis anzutreten. Die Studie bietet mehrere interessante Aspekte zur Diskussion und näheren Betrachtung.

Die Autoren untersuchten erstmalig, inwiefern sich die technischen Vorteile einer MR-Linac-gestützten SBRT im Kontext einer Reduktion des Sicherheitssaums durch eine verbesserte Bildführung und Präzision klinisch auswirken. Die Erfüllung des primären Studienendpunkts hat Signalwirkung und den Autoren darf zur erfolgreichen Planung und Durchführung der Studie gratuliert werden. Die Rate an urogenitalen Grad-≥ 2-Akutnebenwirkungen ist mit 24,4 % niedriger als in der Interventionsgruppe der PACE-B-Studie mit ca. 27 % [4]. Eine vor kurzem veröffentlichte Metaanalyse über 29 Studien mit mehr als 2500 Patienten unterstreicht das Ergebnis der MIRAGE-Studie und konnte eine geringere Rate an urogenitalen und gastrointestinalen Grad-≥ 2-Akutnebenwirkungen im Rahmen der MR-Linac-gestützten SBRT feststellen [5].

Dennoch ist ein genauer Blick auf die Daten und das Design der MIRAGE-Studie angebracht und notwendig. Der primäre Endpunkt umfasste die Inzidenz der höchstgradigen urogenitalen Nebenwirkungen innerhalb der ersten 90 Tage gemäß dem behandelnden Studienarzt. Hier muss betont werden, dass die Studienärzte, welche für die Klassifikation der Nebenwirkungen zuständig waren, nicht verblindet wurden. Durch die Kenntnis des Studienarms kann eine ungewollte Verzerrung bei der Datenerhebung Eingang in die Studie gefunden haben. Die Autoren erkennen dieses Risiko offen an, bemerken aber auch, dass eine Verblindung im Kontext dieser Studie eine signifikante Herausforderung dargestellt hätte. So hätten Patienten im Gespräch ungewollte Informationen nennen können, die Aufschluss über den Behandlungsarm gegeben hätten – hier sei zum Beispiel die invasive Markereinlage vor Bestrahlungsbeginn im Kontrollarm genannt.

Doch nicht nur die Betrachtung des primären Studienendpunkts ist bei dieser Studie von großem Interesse, sondern auch die Daten hinsichtlich der PRO. Hierbei kamen EPIC-26 und IPSS als etablierte Instrumente zur Anwendung. Im experimentellen Studienarm zeigten sich vorteilhafte Ergebnisse u. a. hinsichtlich der Urininkontinenz und bezüglich der gastrointestinalen Domäne. Diese Unterschiede fanden sich jedoch in erster Linie einen Monat nach SBRT. Drei Monate nach Abschluss der Therapie waren die Unterschiede überwiegend marginal, wie auch Siva und Kollegen in ihrem Kommentar betonen [6]. Inwiefern ein anhaltender Nutzen für den Patienten im Kontext der PRO bestehen bleibt, ist unklar. In diesem Kontext muss ebenfalls erwähnt werden, dass die von Studienärzten erhobenen Nebenwirkungen gemäß CTCAE sich durchaus von den PRO unterscheiden können [7].

Die SBRT des PCa hat in den letzten Jahren zunehmend Einzug in die klinische Anwendung gefunden und war Gegenstand zahlreicher Studien [1]. In Zeiten des allgegenwärtigen gesundheitsökonomischen Drucks verbleibt auch die Frage nach dem breitflächigen Implementierungspotenzial einer MR-Linac-gestützten SBRT. Während die aktuellen Gerätekosten noch signifikant sind und der wirtschaftliche Druck für Hersteller der Geräte teilweise zu hoch ist, dürfen z. B. auch zeitliche Ressourcen nicht aus dem Fokus geraten [8–10]. So lag die mediane Behandlungszeit nach durchgeführter Bildgebung für eine Fraktion im experimentellen Arm bei knapp 20 min. Im Kontrollarm lag diese bei unter 5 min. Eine entsprechende Ressourcenbindung hinsichtlich Gerät und Personal gilt es zu beachten [8].

Abschließend ist das onkologische Ergebnis noch ein zentraler Punkt, der betrachtet werden muss. Inwiefern ein reduzierter PTV-Sicherheitssaum mit nur 2 mm trotz der verbesserten bildgestützten Bestrahlung zu einem erhöhten biochemischen Progressionsrisiko nach Primärtherapie führt, ist unklar und kann in Anbetracht der geringen Fallzahl dieser Studie nicht abschließend beantwortet werden [5]. Den Vorteil transienter und reversibler Akuttoxizitäten für eine im Gegenzug unklare Therapieeffektivität aufgrund unzureichender Sicherheitssäume gilt es kritisch zu evaluieren. Die onkologischen Langzeitergebnisse werden sicherlich weitere Erkenntnisse und Diskussionsmaterial liefern [5].

Zusammenfassend ist die MIRAGE-Studie trotz genannter Limitationen ein wichtiger Meilenstein in der Stereotaxie des PCa. Die MR-Linac-gestützte SBRT stellt eine technische Weiterentwicklung dar, die noch viel klinisches Potenzial birgt. Man darf in freudiger Erwartung auf kommende Studien verbleiben – auch wenn noch einige Fragen ungeklärt sind. So ist eine Reduktion der Sicherheitssäume auch mit anderen stereotaxiefähigen Linearbeschleunigern und weiteren technischen Innovationen prinzipiell denkbar [9, 11].

## Fazit

Die MIRAGE-Studie hat ihr Ziel erreicht – die Implementierung der MR-Linac-gestützten SBRT mit einer aggressiven Reduktion von Sicherheitssäumen führt zu einem besseren Nebenwirkungsprofil hinsichtlich der Akuttoxizität bei der Primärbehandlung des PCa. Die Studie nimmt auf dem Gebiet der Prostatastereotaxie einen besonderen Platz ein. Mit Blick auf die Studienlimitationen und die Innovationskraft des MR-Linac bleibt aber die Erkenntnis, dass die strahlentherapeutischen Hausaufgaben noch nicht vollständig gemacht sind. Es bleibt noch viel zu tun, um diesen technischen Fortschritt auch unter gesundheitsökonomischen Aspekten zweifelsfrei in der breiten klinischen Versorgung und mit langfristigem Patientennutzen zu etablieren.

Felix Ehret, Berlin
